# Supplementary material for: Ultra-high performance wearable thermoelectric coolers with less materials
Source: Nat Commun. 2019 Apr 16;10:1765. doi: 10.1038/s41467-019-09707-8 (PMC6468009; doi:10.1038/s41467-019-09707-8)
Supplement: Supplementary file 1 — Supplementary Information [file 41467_2019_9707_MOESM1_ESM.pdf]

Supplementary Information

Ultra-high performance wearable thermoelectric coolers with less materials

Ravi Anant Kishore [1,2,*], Amin Nozariasbmarz [3], Bed Poudel [3], Mohan Sanghadasa [4] and Shashank Priya [1,3,*]

[1] Center for Energy Harvesting Materials and Systems, Virginia Tech, Blacksburg, VA 24061, USA.

[2] National Renewable Energy Laboratory, 15013 Denver West Pkwy, Golden, CO 80401, USA.

[3] Department of Materials Science and Engineering, Pennsylvania State University, University Park, PA 16802, USA.

[4] Aviation and Missile Center, U.S. Army Combat Capabilities Development Command, Redstone Arsenal, AL 35898.

[*] Corresponding authors: Ravi Anant Kishore (ravi86@vt.edu); Shashank Priya (sup103@psu.edu).

Supplementary Note 1: Thermoelectric Effect

Thermoelectric (TE) effect results in direct conversion of thermal energy into electrical energy and vice versa by utilizing the combination of four different phenomena: Joule heating, Seebeck effect, Peltier effect, and Thomson effect[1]. Joule heat or resistive heat is generated when an electric current is passed through an electrical resistor. It is directly proportional to the square of the current and the electrical resistance of the resistor [1,2].

$$Q_j = I^2 R \tag{1}$$

where, Q_j denotes the Joule heat, I is electric current, and R is the electrical resistance.

Seebeck effect results in a voltage difference across the two ends of a thermocouple (made up of p- and n-type TE materials) in response to the applied temperature difference across the two ends of the thermocouple. The induced voltage, called Seebeck voltage, is directly proportional to the temperature difference and is given as[3]:

$$\Delta V = \alpha_{pn} \Delta T \quad (2)$$

where, ΔV is the induced voltage, ΔT is the temperature difference, and α_{pn} denotes the total Seebeck coefficient of the thermocouple.

Peltier effect is reverse of Seebeck effect. It causes cooling or heating at the junction of a thermocouple when an electric current is passed through the junction. The Peltier heat is directly proportional to the current but it is absorbed or released based on the relative direction between the electric current and p-n junction. Peltier heat is mathematically expressed as[1]:

$$Q_p = \pi_{pn} I \quad (3)$$

where Q_p is heating or cooling due to Peltier effect, I is the electric current, and π_{pn} is the Peltier coefficient of the thermocouple.

For most of the TE materials, Seebeck and Peltier coefficients greatly vary with temperature. Therefore, when a spatial gradient in temperature exists in a TE material and an electric current is passed through it, a continuous version of the Seebeck-Peltier effect occurs. This results in Thomson effect. Thomson heat therefore occurs in a non-uniformly heated TE material. Mathematically, Thomson effect is expressed as[1]:

$$\frac{dq}{ds} = \tau I \frac{dT}{ds} \quad (4)$$

where τ is defined as Thomson coefficient of the TE material and s is a spatial coordinate.

For a thermocouple, Seebeck coefficient, α_{pn}, Peltier coefficient, π_{pn}, and Thomson coefficient, τ, are related by equations[2]:

$$\pi_{pn} = \alpha_{pn} T \tag{5}$$

$$\tau_p - \tau_n = T \frac{d\alpha_{pn}}{dT} \tag{6}$$

where

$$\pi_{pn} = \pi_p - \pi_n \tag{7}$$

$$\alpha_{pn} = \alpha_p - \alpha_n \tag{8}$$

where p and n denotes p- and n-type TE materials of the thermocouple.

Supplementary Note 2: Thermoelectric cooler (TEC)

Thermoelectric cooler (TEC) is a solid-state heat pump that consume electricity to produce heating or cooling effect. Technically, TECs can be used for both heating and cooling purposes, but they are primarily used in the cooling mode. Traditionally, there are two ways to quantify the TEC performance. In the first method, temperature on the two sides of TEC is fixed while a variable electric current is passed through it. The maximum heat transfer rate from cold-side of TEC is recorded, which is defined as cooling capacity (Q_c) of the TEC at that temperature difference. In the second method, hot-side of TEC is fixed at a certain temperature and a constant heat flux is passed through the cold-side. The cold-side temperature is then monitored while a variable electric current is applied. The maximum temperature difference between the two sides of TEC is recorded, which is defined as ΔT capacity of the TEC at that heat load. Both these approaches are

interchangeability used in the literature for calculating TEC performance[4-13]. For single stage commercial TECs, a maximum temperature difference of about 70°C is a widely reported value, when hot-side is fixed at 27°C[14,15]. Since human body is more sensitive to temperature change, we have used temperature drop (ΔT capacity) is this study to characterize TECs for body cooling.

Coefficient of performance (COP) is another very important performance metric of TECs. COP provides cooling capacity per unit input electrical power. In the similar fashion, we can also measure ΔT capacity per unit input electrical power. This parameter (cooling over input power) has been used in this study to compare different fill factor TEC modules. Sometimes, it is also necessary to compare cooling over volume of the TE material. Since TE materials, particularly high efficiency nanostructured materials, are expensive, this factor gives an idea about the amount of material (and thus material cost) needed to achieve same amount of cooling. This parameter (cooling over material volume) has been used in this study.

Supplementary Table 1. Geometric parameters, material properties, and other features of the four thermoelectric cooler (TEC) models

	**TEC 1**	**TEC 2**	**TEC 3**	**TEC 4**
Leg height (mm)	1.6	1.6	1.6	1.6
Leg cross-sectional area (mm^2)	1.6 × 1.6	1.6 × 1.6	1.6 × 1.6	1.6 × 1.6
Fill factor	25%	25%	25%	25%
Seebeck coefficient (μV/K)	177	250	177	177
Electrical conductivity (S/m)	10^5	10^5	2.0×10^5	10^5
Thermal conductivity (W/m-K)	1.5	1.5	1.5	0.75
zT at 25°C	0.62	1.25	1.25	1.25
Electrical contact resistivity (Ω-m^2)	10^{-9}	10^{-9}	10^{-9}	10^{-9}
Electrode material	Cu	Cu	Cu	Cu
Electrode thickness (mm)	0.2	0.2	0.2	0.2
Substrate material	AlN	AlN	AlN	AlN

Supplementary Table 2. Typical values of heat transfer coefficient (h)[16,17]

Type of convection	h ($Wm^{-2}K^{-1}$)
Free convection of gas	2-25
Free convection of liquid	10-1000
Forced convection of gases	25-250
Forced convection of liquids	50-20,000
Boiling and condensation	2500-100,000
Human skin	20-100

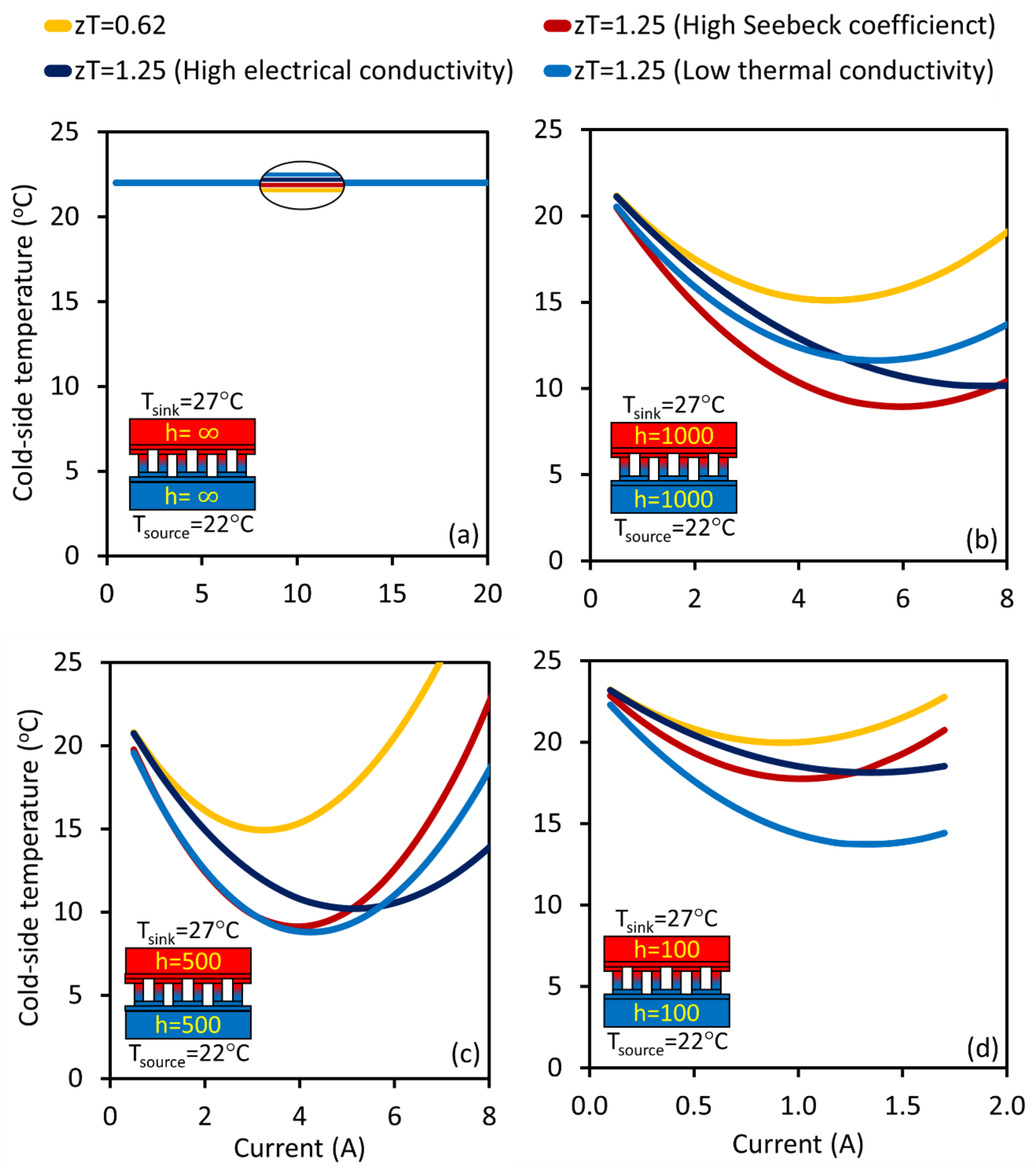

Supplementary Fig. 1. Effect of thermally resistive environment on cold-side temperature. Although zT value is same, the effect of material properties on cold-side temperature changes with change in thermal resistance of heat source/sink. (a) When heat source resistance is zero, cold-side temperature is equal to the core temperature of the heat source and thus it does not change with change in material properties. (b) When source/sink resistances are low (h = 1000 $Wm^{-2}K^{-1}$), TE

materials with higher Seebeck coefficient results in minimum cold-side temperature. (c) The effect of thermal conductivity on cooling increases with increase in source/sink thermal resistances. (d) When heat source and sink resistances are very high ($h = 100$ $Wm^{-2}K^{-1}$), low thermal conductivity results in highest cooling and thus least cold-side temperature.

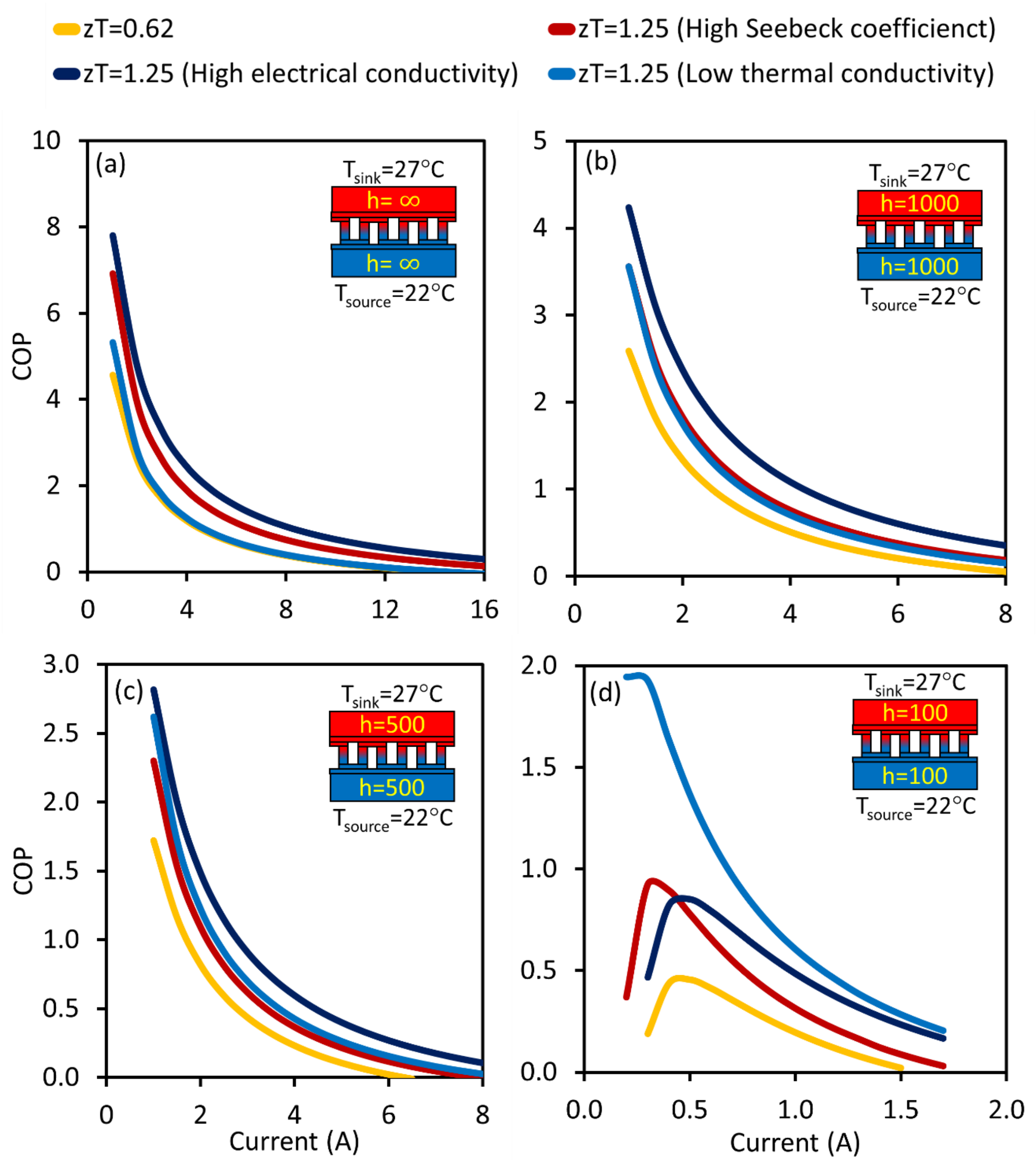

Supplementary Fig. 2. Effect of thermally resistive environment on COP. Effect of material properties on COP is different in the different environmental condition, even if zT value is same. (a) Electrical conductivity has strongest effect on COP, when heat source/sink resistances are low (h = 1000 Wm^{-2}K^{-1}). (b)-(c) The effect of thermal conductivity on COP increases with increase

in source/sink thermal resistances. (d) When heat source and sink resistances are very high ($h = 100$ $Wm^{-2}K^{-1}$), low thermal conductivity results in highest COP.

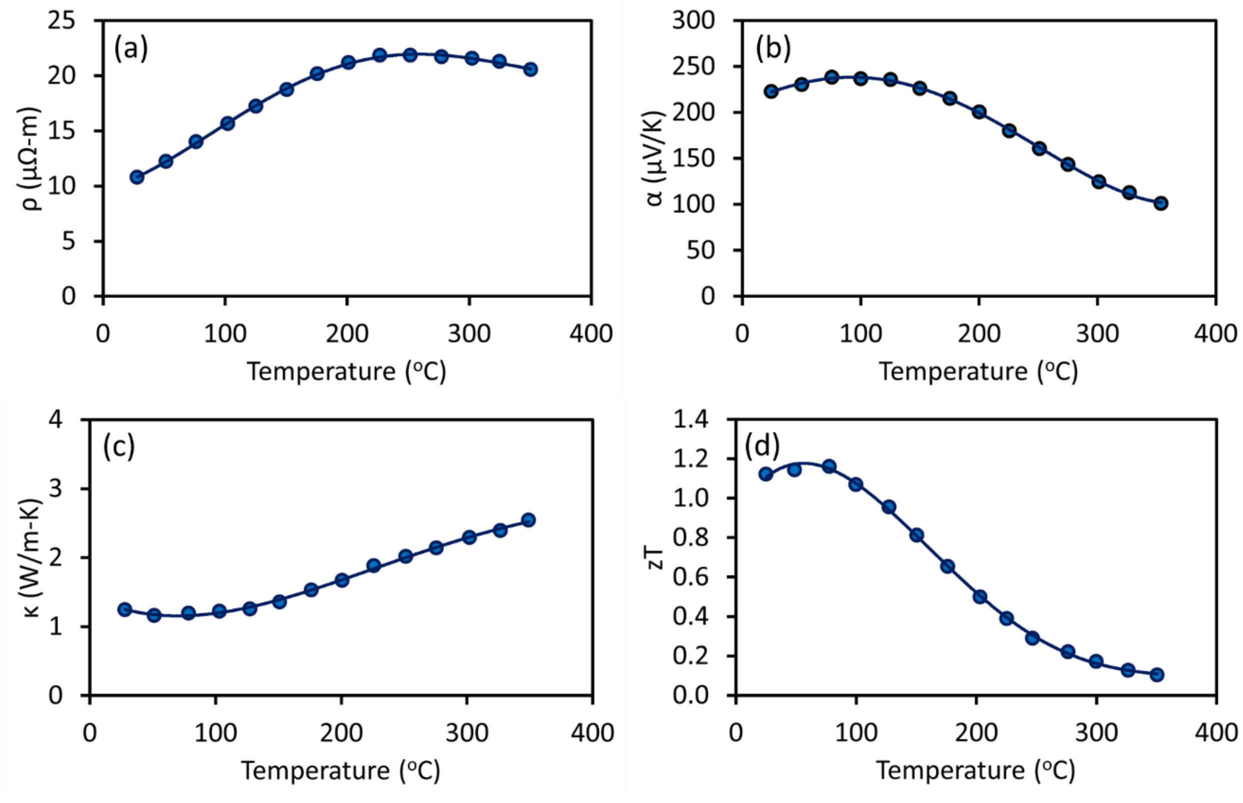

Supplementary Fig. 3. Material properties of commercial p-type bismuth telluride. (a) Electrical resistivity (ρ), (b) Seebeck coefficient (α), (c) Thermal conductivity (κ) and (d) zT. Information taken from reference:[18].

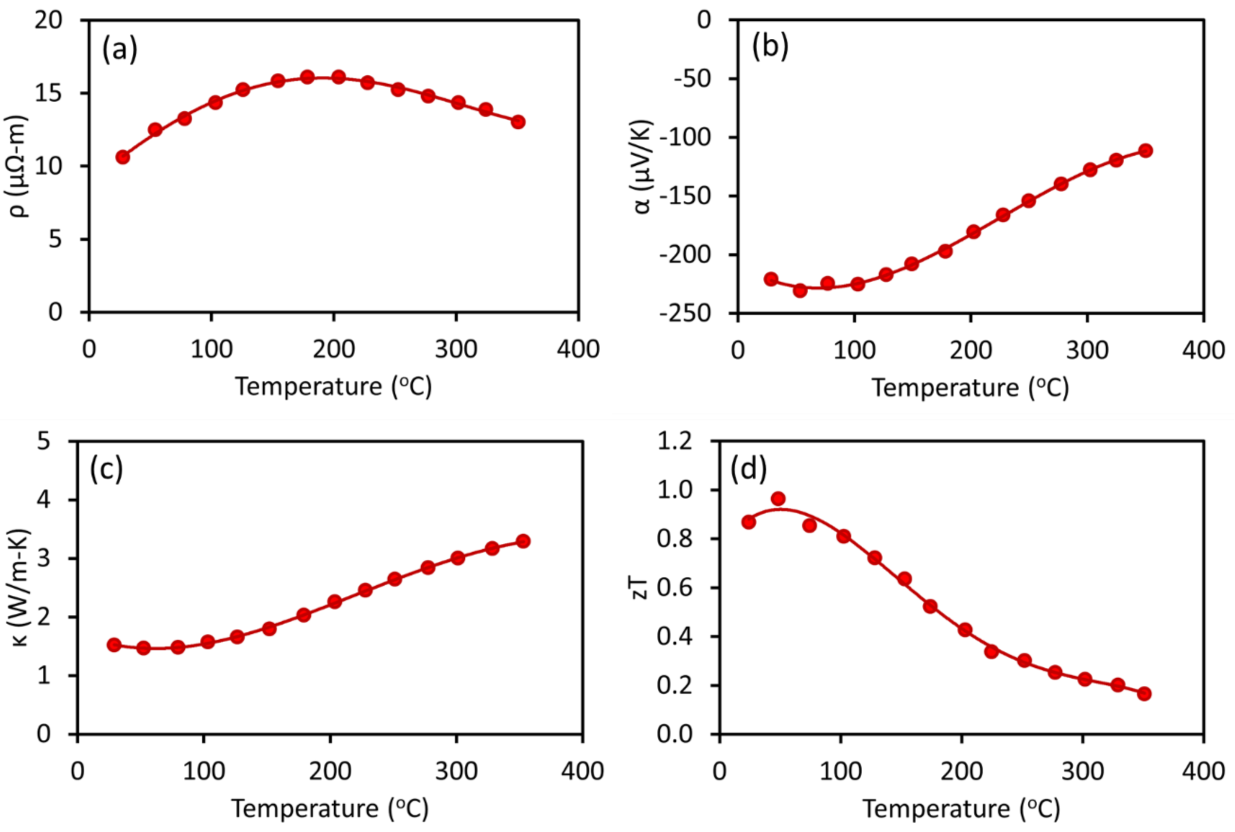

Supplementary Fig. 4. Material properties of commercial n-type bismuth telluride. (a) Electrical resistivity (ρ), (b) Seebeck coefficient (α), (c) Thermal conductivity (κ) and (d) zT. Information taken from reference:[18].

Supplementary Note 3: Optimal module design for wearable TECs

In the normal ambient conditions (~27°C), the human skin temperature at forehead is ~35.2°C and it slowly decreases as we move down towards the foot (~30.4°C)[19]. As the ambient atmospheric condition changes, the skin temperature changes as well, giving sensation of hotness or coldness. For example, in extreme summer when ambient temperature is 47°C, the temperature at forehead rises to 37°C; whereas in winter, when ambient temperature is 15°C, the temperature at forehead reduces to 31.7°C[19]. Human skin, by natural evolution, is a poor conductor of heat. Depending on the physiological parameters such as weight, age, body fat and even gender the skin resistance can greatly vary [20]. The convective heat transfer coefficient of the human skin has been reported to be around 5 $Wm^{-2}K^{-1}$ [21]. In addition, after a TEC is deployed on the human body, the contact resistance at the TEG-skin interface is strongly dependent on the interface material and the contact pressure. The effective heat transfer coefficient (h_{skin}) for the human skin under TE module has been reported to lie in the range of 20-100 $Wm^{-2}K^{-1}$ [17]. For a rigid TEG deployed on a human forearm, Suarez et al. used $h_{skin} = 50$ $Wm^{-2}K^{-1}$ [22].

On the hot-side, the wearable TECs usually require fins as the heat sink to augment the heat rejection. The effective thermal resistance of the heat sink containing an array of fins is given as:[23]

$$R_{sink} = \frac{1}{\eta_o h A_t} \tag{9}$$

where η_o is the overall efficiency of the fins and A_t is the total surface area exposed portion for cooling (i.e., $A_t = mA_f + A_b$, where m is number of fins, A_f is surface area of a fin and A_b is the exposed portion of the base). Considering $\eta_o = 95\%$ and $h_{air} = 5$ $Wm^{-2}K^{-1}$, the effective heat transfer coefficient for an aluminum heat sink containing fins of dimension 10 mm × 10 mm × 0.5 mm can be found to be $h_{sink} = 100$ $Wm^{-2}K^{-1}$.

It can be noted that due to high thermal resistivity of the human skin and the ambient air along with the size constraint of the heat sink for on-body applications, wearable TECs operate in extremely high thermally resistive environment. This is evident from the heat transfer coefficient of human skin and heat sink, which is less than 100 $Wm^{-2}K^{-1}$. The TE material composition and module design for on-body applications are, therefore, quite different from the typical commercial modules. The TEC modules deployed for the typical applications, where external (heat source and heat sink) thermal resistances are low, the TE materials with high zT and large Seebeck coefficient and electrical conductivity are desired. However, for the applications where heat source and heat sink resistances are large, such as human body, the TE material with low thermal conductivity is required. In this study, in order to minimize the cost, we have utilized commercial bismuth telluride TE materials for fabricating the different fill factor TEC modules. Thermal conductivity of the TE materials used in the study is ~1.25 $Wm^{-1}K^{-1}$ for p-type and ~1.5 $Wm^{-1}K^{-1}$ for n-type at 30°C. Seebeck coefficient at 30°C is ~ 220 μVm^{-1} and electrical resistivity at 30°C is ~10.8 μΩ-m for both p- and n-type materials. Collectively, the material zT at 30°C was found to be 1.1 for p-type and 0.87 for n-type TE materials. The key dimensional features of TEC modules used this study are provided in Supplementary Table 3. The high FF TEC module contains 18 leg pairs, whereas the low FF TEC and the ultra-low FF TEC modules contain 6 leg pairs. The dimensions of p- and n-type legs are 1.6 mm (length) × 1.6 mm (width) × 1.6 mm (height) for the high and low FF TECs, whereas the ultra-low FF TEC contains legs of dimensions: 1.05 mm (length) × 1.05 mm (width) × 1.6 mm (height). The base area of all the TEC modules is 16 mm × 16 mm. This infers that the high FF TEC has fill factor of 36% and leg aspect ratio of 1.0, the low FF TEC has fill factor of 12% and leg aspect ratio of 1.0, and the ultra-low FF TEC has fill factor of 5.2% and leg aspect

ratio of 1.6. The internal electrical resistance measured using four probe method was found to be 228 mΩ, 75 mΩ, and 185 mΩ for high FF, low FF, and ultra-low FF TEC modules, respectively.

Supplementary Table 3. The key features of the fabricated TEC modules

**Description**	**High FF**	**Low FF**	**Ultra-low FF**
Module area (mm^2)	16 × 16	16 × 16	16 × 16
Leg height (mm)	1.6	1.6	1.6
Leg base area (mm^2)	1.6 x 1.6	1.6 x 1.6	1.05 x 1.05
Fill factor	36%	12%	5.2%
Electrode material	Cu	Cu	Cu
Electrode thickness (mm)	0.14	0.14	0.14
Substrate material	AlN	AlN	AlN
Substrate thickness (mm)	0.64	0.64	0.64
TEC electrical resistance (mΩ) at room temperature	228	75	185

Supplementary Note 4: TEC area and weight on human body

It has been suggested that if a localized thermal management system is able to remove 23 W of heat from human body, the cooling setpoint of household heating, ventilation, and air conditioning (HVAC) system can be increased by 2°C, leading to considerable saving in energy consumption [24]. Supplementary Table 4 illustrates the calculations for the TEC area needed for the removal of 23 W of body heat. It can be noted that low and ultra-low FF TECs require ~180 cm^2 of the skin coverage, which is ~1% of the human body. In addition, Supplementary Table 5 below illustrates the calculations for the TEC weight (including heat sink) needed for the removal of 23 W of body heat. It can be noted that low and ultra-low FF TECs weigh ~500 gm, which is within the range of the weight of clothes and other day-to-day wearables.

Supplementary Table 4. Calculations illustrating TEC area needed for 23 W of body heat removal

	TEC base area (cm^2)	Avg. cooling (mW/cm^2)	TEC area needed for 23 W (cm^2)	% of an adult human body
High FF TEC	2.56	97.5	235.9	~1.31%
Low FF TEC	2.56	130.0	176.9	~0.98%
Ultra-low FF TEC	2.56	122.5	187.7	~1.04%

Supplementary Table 5. Calculations illustrating TEC weight for 23 W of body heat removal

	Weight of TEC with heat sink (gm)	TEC base area (cm^2)	Avg. cooling (mW/cm^2)	Weight of TECs for 23 W of cooling (gm)
High FF TEC	7.9	2.56	97.5	728
Low FF TEC	7.1	2.56	130.0	491
Ultra-low FF TEC	6.9	2.56	122.5	506

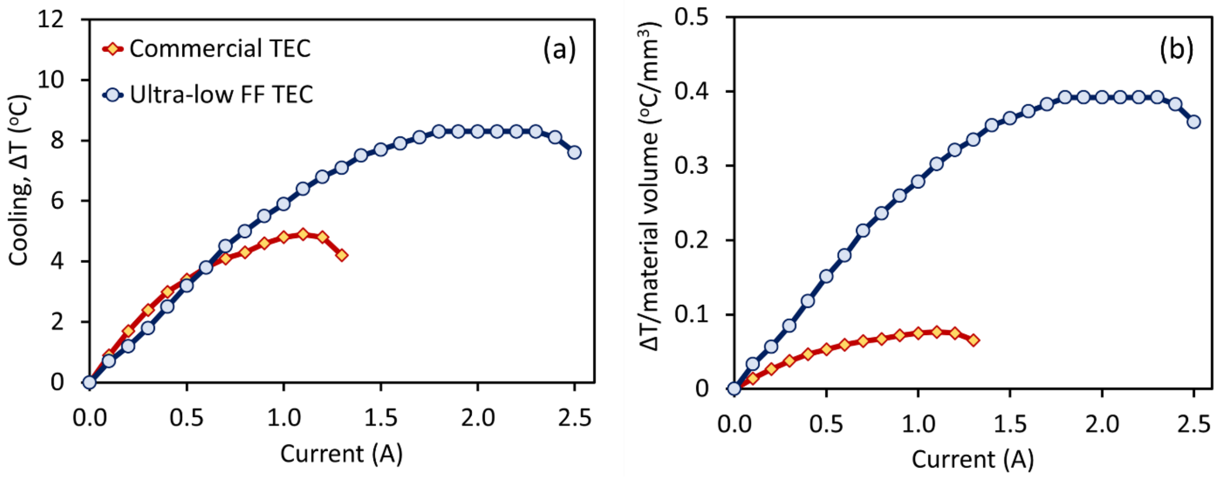

Supplementary Fig. 5. Comparison between the commercial TEC and ultra-low FF TEC. (a) Cooling versus electric current. (b) Cooling over volume of the TE materials versus electric current.

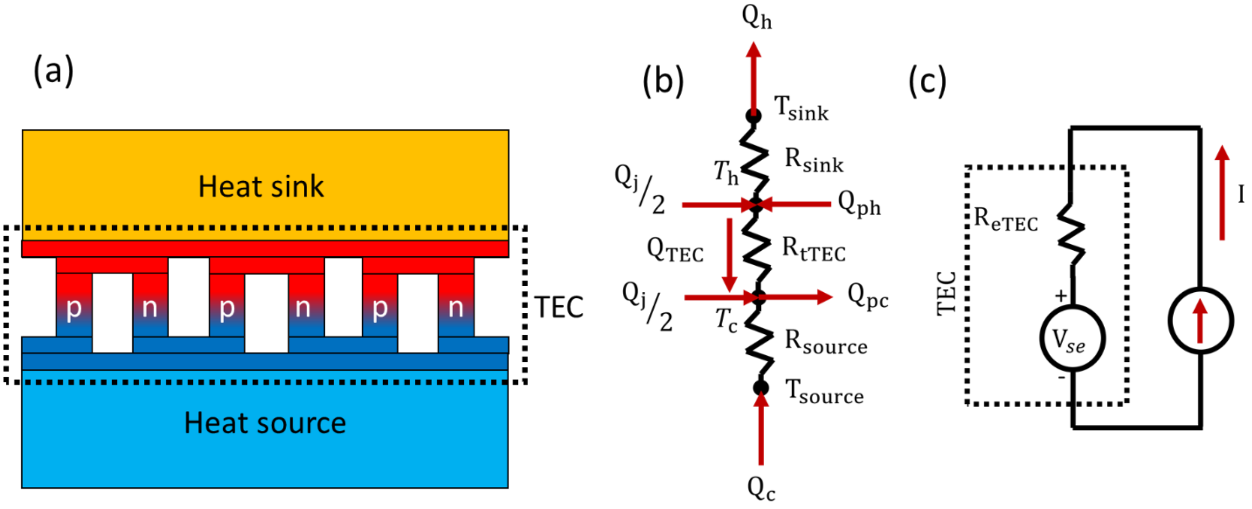

Supplementary Fig. 6. Simplified one-dimensional energy equilibrium model of a TEC. (a) Schematic illustration of the structure of a TEC, which consists of several p- and n-type legs connected electrically in series and thermally in parallel. Heat source and heat sink on the two sides of TEC can create a thermally resistive environment for TEC, affecting its design and performance. (b) One-dimensional thermal circuit illustrating the heat flow from heat source to heat sink via TEC. Performing energy balance on hot- and cold-sides provides mathematical relations that can be solved to obtain cooling capacity and the coefficient of performance (COP). (c) The electrical-circuit of TEC depicting the Seebeck voltage and internal electrical resistance.

Supplementary references

1 Kishore, R. A., Kumar, P. & Priya, S. A comprehensive optimization study on Bi 2 Te 3-based thermoelectric generators using the Taguchi method. *Sustainable Energy Fuels* **2**, 175-190 (2018).

2 Kishore, R. & Priya, S. A Review on Low-Grade Thermal Energy Harvesting: Materials, Methods and Devices. *Materials* **11**, 1433 (2018).

3 Ko, S. H. & Grigoropoulos, C. P. *Hierarchical nanostructures for energy devices*. (Royal Society of Chemistry, 2014).

4 Lin, S. & Yu, J. Optimization of a trapezoid-type two-stage Peltier couples for thermoelectric cooling applications. *Int. J. Refrig* **65**, 103-110 (2016).

5 Cheng, Y.-H. & Shih, C. Maximizing the cooling capacity and COP of two-stage thermoelectric coolers through genetic algorithm. *Appl. Therm. Eng.* **26**, 937-947 (2006).

6 Melnikov, A., Kostishin, V. & Alenkov, V. Dimensionless Model of a Thermoelectric Cooling Device Operating at Real Heat Transfer Conditions: Maximum Cooling Capacity Mode. *J. Electron. Mater.* **46**, 2737-2745 (2017).

7 Anant Kishore, R., Kumar, P., Sanghadasa, M. & Priya, S. Taguchi optimization of Bismuth-Telluride based thermoelectric cooler. *J. Appl. Phys.* **122**, 025109 (2017).

8 Lee, K. H. & Kim, O. J. Analysis on the cooling performance of the thermoelectric micro-cooler. *Int. J. Heat Mass Transfer* **50**, 1982-1992 (2007).

9 Zhou, Y. & Yu, J. Design optimization of thermoelectric cooling systems for applications in electronic devices. *Int. J. Refrig* **35**, 1139-1144 (2012).

10 Min, G. & Rowe, D. Improved model for calculating the coefficient of performance of a Peltier module. *Energy Convers. Manage.* **41**, 163-171 (2000).

11 Huang, Y.-X., Wang, X.-D., Cheng, C.-H. & Lin, D. T.-W. Geometry optimization of thermoelectric coolers using simplified conjugate-gradient method. *Energy* **59**, 689-697 (2013).

12 Marlow. *Thermoelectric cooler: https://www.marlow.com/products/thermoelectric-coolers. Accessed on June 16, 2018.*

13 Ferrotec. *General-Purpose Thermoelectric Cooler Modules: https://thermal.ferrotec.com/products/peltier-thermoelectric-cooler-modules/general-purpose/. Accessed on June 16, 2018*.

14 Datasheet1. *Technical Data Sheet for CM23-1.9. https://cdn2.hubspot.net/hubfs/547732/Data_Sheets/CM23-1.9.pdf. Accesses on June 10, 2018.*

15 Datasheet2. *ATE1-07 TEC Modules.*

http://www.analogtechnologies.com/document/ATE1-07.pdf. Accesses on June 10, 2018.

16 Cengel, Y. *Heat and mass transfer: fundamentals and applications*. (McGraw-Hill Higher Education, 2014).

17 Lossec, M., Multon, B., Ahmed, H. B. & Goupil, C. Thermoelectric generator placed on the human body: system modeling and energy conversion improvements. *The European Physical Journal-Applied Physics* **52** (2010).

18 Hu, X. *et al.* Power generation from nanostructured PbTe-based thermoelectrics: comprehensive development from materials to modules. *Energy Environ. Sci.* **9**, 517-529 (2016).

19 Webb, P. Temperatures of skin, subcutaneous tissue, muscle and core in resting men in cold, comfortable and hot conditions. *European journal of applied physiology and occupational physiology* **64**, 471-476 (1992).

20 Huizenga, C., Hui, Z. & Arens, E. A model of human physiology and comfort for assessing complex thermal environments. *Build. Environ.* **36**, 691-699 (2001).

21 Kurazumi, Y. *et al.* Radiative and convective heat transfer coefficients of the human body in natural convection. *Build. Environ.* **43**, 2142-2153 (2008).
22 Suarez, F., Nozariasbmarz, A., Vashaee, D. & Öztürk, M. C. Designing thermoelectric generators for self-powered wearable electronics. *Energy & Environmental Science* **9**, 2099-2113 (2016).
23 Bergman, T. L., Incropera, F. P., DeWitt, D. P. & Lavine, A. S. *Fundamentals of heat and mass transfer*. (John Wiley & Sons, 2011).
24 ARPA-E. *ARPA-E DELTA Program Overview:* https://arpa-e.energy.gov/sites/default/files/documents/files/DELTA_ProgramOverview.pdf*. Accessed on Jan 16, 2019.*
